# Structural Validity of a Short Form of the Eating Disorder Examination Questionnaire in Adolescents Diagnosed With an Eating Disorder

**DOI:** 10.1002/eat.70146

**Published:** 2026-06-15

**Authors:** Paul E. Jenkins, Sasha Gorrell, Erin C. Accurso, Sarah Eckhardt, Elizabeth K. Hughes, Daniel Le Grange, Susan M. Sawyer, Renee D. Rienecke

**Affiliations:** ^1^ School of Psychology and Clinical Language Sciences, University of Reading Reading UK; ^2^ Department of Psychiatry and Behavioral Sciences University of California San Francisco California USA; ^3^ Philip R. Lee Institute for Health Policy Studies University of California San Francisco San Francisco California USA; ^4^ Center for the Treatment of Eating Disorders, Children's Minnesota St Paul Minnesota USA; ^5^ Department of Paediatrics The University of Melbourne Parkville Victoria Australia; ^6^ Murdoch Children's Research Institute Parkville Victoria Australia; ^7^ Centre for Adolescent Health, Royal Children's Hospital Parkville Victoria Australia; ^8^ Department of Psychiatry and Behavioral Sciences Northwestern University Chicago Illinois USA

**Keywords:** assessment, eating disorder examination‐questionnaire, latent variable analysis, patient‐reported outcome measures, psychometrics, readability

## Abstract

**Objective:**

This study aimed to assess the structural validity and readability of a brief 7‐item version of the Eating Disorder Examination Questionnaire (the EDE‐Q7) among adolescents.

**Method:**

Latent variable analysis was used in a sample of 263 adolescents (age range = 13–17.99y) diagnosed with an eating disorder and recruited from treatment centres in the United States of America and Australia. Several indices were used to provide different estimates of overall model fit.

**Results:**

The EDE‐Q7 showed good fit to the data, although absolute fit indices suggested some evidence of imperfect fit. Several other models (both brief and full‐item versions) failed to meet most criteria indicating good fit. The subscales of the EDE‐Q7 showed significant correlations with several disordered eating behaviors, suggesting convergent validity. Review of the EDE‐Q7 suggests ease of readability for adolescents from at least 11 years of age.

**Discussion:**

This is one of the first clinical studies to support the EDE‐Q7 as a brief, valid assessment of eating psychopathology that can be used with adolescents as well as adults. The results indicate that the EDE‐Q7 might be appropriate for assessing eating pathology among adolescents from the age of 13 years.

## Summary

- This study addresses a critical gap in eating disorder research by validating a brief version of the Eating Disorder Examination Questionnaire (the EDE‐Q7) in a sample of adolescents.
- Compared to most other models, the EDE‐Q7 showed good validity and was deemed to be fairly easy to read, appropriate for most young people over the age of 11.
- Demonstrating the appropriateness of the EDE‐Q in this population will help evaluate treatments in this age group and allow comparison of symptoms.

## 1 Introduction

The Eating Disorder Examination Questionnaire (EDE‐Q; Fairburn and Beglin 1994, 2008) is an established self‐report measure of disordered eating attitudes and behaviors. Twenty‐two items assess the attitudinal symptoms of eating problems with a further six assessing the frequency of several disordered eating behaviors, such as objective binge eating and self‐induced vomiting. Reflecting the cognitive‐behavioral theory of eating disorders (e.g., see Cooper et al. 1989; Fairburn 2008), the EDE‐Q is often conceptualized as comprising four subscales: (Dietary) Restraint, Eating Concern, Shape Concern, and Weight Concern. Commonly used in adult populations, the extent to which the EDE‐Q is appropriate for use in adolescents is unknown. For instance, modifications to the wording of the EDE‐Q have been proposed, as well as a reduction in the timeframe assessed by the measure from 28 to 14 days—suggestions originally based on the developmental appropriateness of the items and clinical experience in the assessment of eating pathology (see Carter et al. 2001). In addition, although aspects of the reliability and validity of the EDE‐Q have been supported in studies of adults (e.g., Berg et al. 2012), there is less psychometric data in samples of adolescents with eating problems from which to draw robust conclusions. The unmodified (i.e., ‘original’) version of the EDE‐Q has been used in large studies of adolescents (e.g., Mond et al. 2014) and similar measures using shorter timeframes in youth correlate strongly with this version (e.g., Accurso and Waller 2021). Establishing consonance of the ‘unmodified’ EDE‐Q in adolescents therefore has the advantage of enabling usage across the age range and promoting consistency.

Where data exist on adolescent samples (e.g., Berg et al. 2013), patterns regarding the psychometric performance of the EDE‐Q have mirrored the inconsistencies found in adults. For instance, several studies have failed to replicate the proposed four‐factor structure of the 22 attitudinal items of the EDE‐Q in both adolescents (e.g., Penelo et al. 2013) and adults (see Jenkins and Rienecke 2022, for a review), undermining conclusions regarding the reliability of this solution for accurately assessing eating psychopathology. Studies using exploratory factor analysis (EFA) in young people have resulted in several proposed solutions, all of which differ from the original version (Fairburn and Beglin 2008); these have included the EDE‐Q modified for adolescents (e.g., Forsén Mantilla et al. 2017) and the original version (e.g., Mitsui et al. 2017; White et al. 2014). Subsequent confirmatory factor analyses (CFA) in different samples, whilst limited in scope, have failed to find clear support for the interpretations proposed by EFA (e.g., Penelo et al. 2013; Sepúlveda et al. 2019). Overall, findings from adolescent studies generally align with those from adult samples, whereby the four‐factor structure of the EDE‐Q has not been reliably supported (see Jenkins and Rienecke 2022).

The lack of consistency and absence of a clearly supported factor structure pose problems for the administration, scoring, and interpretation of the EDE‐Q. For instance, uncertainty regarding the structural validity of the EDE‐Q constrains the ability to compare scores among different groups (e.g., genders, cultures; see Steenkamp and Baumgartner 1998), and hinders the interpretation of resulting scores. Further, investigating structural validity, a type of construct validity, can add insight into the latent construct, such as how much variance is accounted for by an individual item and how much is shared across items, and thus ‘explained’ by a construct.

In contrast to “full‐item” versions of the EDE‐Q (i.e., those including all 22 attitudinal items of the EDE‐Q), a brief version consisting of seven items (EDE‐Q7; Grilo et al. 2013) has shown evidence of structural validity in adult samples, including those from a variety of backgrounds and cultures (see Jenkins and Rienecke 2022). The EDE‐Q7 offers several advantages over the longer version, including reduced administration time and omission of several items which have proven ‘troublesome’ in some groups (e.g., an item assessing prescribed weighing; Darcy et al. 2013). Early support for use of the EDE‐Q7 with adolescents comes from a study using a translated version of this measure in a community sample recruited from 10 girls' schools and nine boys' schools across Iran (Sahlan et al. 2023). Comparing several existing models of the EDE‐Q, this study found that only the 7‐item model of Grilo et al. (2013) provided good fit to the data, with consistently strong factor loadings (suggesting good “explanatory power” of the latent variable). Furthermore, the EDE‐Q7 demonstrated convergent validity and showed that meaningful comparisons can be made across gender (an example of “scalar invariance”; see Steenkamp and Baumgartner 1998). A study of a young Mexican clinical sample (93.1% female; 88.4% aged between 11 and 25 years) tested a range of EDE‐Q models using CFA (Trujillo‐ChiVacuán et al. 2025). Similar to the findings of Sahlan et al. (2023), the 7‐item model of Grilo et al. (2013) showed “superior fit indices compared to other tested models” (p. 9).

There have been few empirical investigations of the EDE‐Q7's factor structure in clinical adolescent samples using the timeframe and wording originally suggested. Such studies could offer insight into the optimal use of the EDE‐Q7 and its applicability in younger samples and may suggest what modifications, if any, are needed on account of age. The “readability” of the EDE‐Q has been estimated at around the 8th—9th grade level (approximately ages 13–15) (Richards et al. 2013) and, whilst this is acknowledged to be only one component of comprehension, suggests that older adolescents will typically find reading the text relatively easy (see also Richards et al. 2013). Comparing several interpretations of the EDE‐Q's factor structure derived from diverse community samples of adolescents (e.g., Penelo et al. 2013; Lewis‐Smith et al. 2021; White et al. 2014) would extend findings to clinical samples, in addition to investigating further the structural validity of different models (e.g., Trujillo‐ChiVacuán et al. 2025).

The current study aimed to address these gaps by investigating the readability and structural validity of the EDE‐Q7 alongside several different models, including both full‐item and brief versions. To do so, we included a clinical sample of adolescents diagnosed with a range of eating disorders aged over 13 years, a figure determined based on the readability of the EDE‐Q, as well as cutoffs used in previous studies (e.g., Forrest et al. 2017; Lock 2015). Given previous work in non‐clinical samples of adolescents (e.g., Trujillo‐ChiVacuán et al. 2025; Sahlan et al. 2023), we hypothesized that the EDE‐Q7 would fit the data well using CFA, including when compared to other models. Model choice was informed by existing work; two found to perform well in adult samples (Friborg et al. 2013 [‘Friborg’]; Peterson et al. 2007 [‘Peterson’]; see Jenkins and Rienecke 2022) were included, as well as three with some support in adolescent samples (Lewis‐Smith et al. 2021 [“Lewis‐Smith”]; Penelo et al. 2013 [“Penelo”]; White et al. 2014 [“White”]). In addition, a Canadian study including older adolescents and adults (Dufour et al. 2025) suggested that a one‐factor, five‐item measure [“Dufour”] might be useful as an alternative brief form. These models are summarized in Table 1.

**TABLE 1:** Summary of models evaluated in the study.

Model (authors)	Summary of sample (context, gender, age, country)	Factors	Items (numbers drawn from Fairburn and Beglin 2008)
*Full‐item models*			
Fairburn and Beglin (2008) [“Original”]	Community sample of 243 young women (aged 16–35 years), United Kingdom (see Fairburn and Beglin 1994, for details)	(Dietary) Restraint Eating Concern Shape Concern Weight Concern	1–5 7, 9, 19–21 6, 8, 10, 11, 23, 26–28 8, 12, 22, 24, 25
Friborg et al. (2013) [“Friborg”]	Representative community sample of 1076 women (aged 16–50y), Norway	S1 S2 S3 S4	6, 10, 11, 12, 22–28 2, 5, 7, 8, 9 1, 3, 4 19–21
Penelo et al. (2013) [“Penelo”]	Regional school sample of 2928 students (53% female, aged 11–18 years), Mexico	Restraint Eating, Weight, Shape Concerns	1–5 6–12, 19– 28
Peterson et al. (2007) [“Peterson”]	Community sample of 203 women (aged 18–57 years), United States of America	Weight and Shape Concerns Eating Concern Restraint	6, 10, 11, 12, 22–28 5, 7, 8, 9, 19– 21 1– 4
White et al. (2014) [“White”]	Community sample of 917 adolescents (57% female, aged 14–18 years), United Kingdom	Shape and Weight Concerns Restriction Preoccupation and Eating Concern	6, 11, 12, 22– 28 1, 3– 5, 10 2, 7– 9, 19– 21
*Reduced‐item (“brief”) models*			
Dufour et al. (2025) [“Dufour”]	1197 patients from a mental health research centre (95% female, aged 15–65 years), Canada		10– 12, 22, 23
Grilo et al. (2015) [“EDE‐Q7”]	801 university students (72% female, aged 18–47 years), United States of America	Dietary Restraint Overvaluation of Weight and Shape Body Dissatisfaction	1, 3, 4 22, 23 25, 26
Lewis‐Smith et al. (2021) [“Lewis‐Smith”]	1413 secondary school students (45% female; aged 11–15 years), India	Preoccupation Weight and Shape Concerns	1, 4– 7, 9 20, 21, 24– 27

*Note:* The factor solution from Lewis‐Smith et al. (2021) was derived from the full sample, and slightly different solutions were reported for boys and girls when treated as independent samples.

## 2 Method

### 2.1 Participants and Procedures

The current data were drawn from four clinical studies of individuals diagnosed with an eating disorder according to DSM‐5 criteria (American Psychiatric Association 2013) who presented to three treatment sites across the continental United States of America (USA) or Australia (*N* = 1509) between 2010 and 2019. The Institutional Review Boards at each participating institution gave ethics approval for the ongoing use of data within data use agreements. To maintain relevance to the research question of this study, which reports secondary outcome analyses of data collected across several outpatient care settings, the sample (*N* = 263; *n* = 158 from US; *n* = 105 from Australia; 13% male) was limited to individuals between the ages of 13–17.99 years old (*M*[SD] = 15.50[1.37]) who met diagnostic criteria for anorexia nervosa, bulimia nervosa, binge‐eating disorder, or their atypical forms. Individuals with a diagnosis of Avoidant/Restrictive Food Intake Disorder were excluded. Of those with reported race and ethnicity from the USA (*n* = 156), a minority were non‐White or Hispanic (see Table 2 for demographic details). Participants recruited in Australia (~40% of the sample) did not have race or ethnicity reported.

**TABLE 2:** Sociodemographic characteristics of participants.

Characteristic	USA sample (*n* = 158)	Australia sample (*n* = 105)	Total sample (*n* = 263)
Age, *M* (SD)	15.47 (1.38)	15.56 (1.36)	15.50 (1.37)
	*n* (%)	*n* (%)	*n* (%)
Diagnosis			
AN	78 (49.4)	64 (61)	142 (53.9)
BN	18 (11.4)	2 (1.9)	20 (7.6)
BED	5 (3.2)	1 (1.0)	6 (2.3)
Atypical AN	49 (31.0)	38 (36.2)	87 (33.1)
Atypical BN	6 (3.8)		6 (2.3)
Atypical BED	2 (1.3)		2 (0.7)
Sex, female	131 (82.9)	97 (92.4)	228 (86.7)
Race			
White	126 (79.7)		
Mixed or Other	17 (10.9)		
Asian	11 (7.0)		
Black or African American	1 (0.6)		
American Indian or Alaska Native	1 (0.6)		
Not reported	2 (1.3)		
Ethnicity			
Hispanic/Latino	21 (13.3)		
Non‐Hispanic/Latino	135 (85.4)		
Not reported	2 (1.3)		

*Note:* Data for race and ethnicity were not collected in the Australia sample.

Abbreviations: AN, anorexia nervosa; BED, binge‐eating disorder; BN, bulimia nervosa.

### 2.2 Measures

All participants completed the EDE‐Q v6.0 (Fairburn and Beglin 2008), a 28‐item, self‐report instrument designed to assess behaviors and concerns regarding eating, weight, and shape over the past 4 weeks (28 days); no modifications to the questionnaire were made. Twenty‐two items assessing disordered attitudes are typically scored based on four subscales: (Dietary) Restraint, Eating Concern, Weight Concern, and Shape Concern, with a Global score calculated from the average of these subscales. Six further items, scored on a ratio scale, assess disordered eating behaviors and are typically excluded from estimation of latent variables; they are used here to assess convergent validity. Cronbach's alpha across the 22 items suggested good internal consistency in the current sample (*α* = 0.87). All participants had at least some EDE‐Q data, with overall missingness of 5.7% of EDE‐Q items.

### 2.3 Statistical Analyses

#### 2.3.1 Readability

To assess readability of the EDE‐Q7, the R package *sylcount* (version 0.2‐6; Schmidt 2023) was used. By basing ratings on the average number of syllables per word and words per sentence, both the Flesch Reading Ease Test and the Flesch–Kincaid Grade Level Test can be estimated. The Flesch Reading Ease Test is rated on a 100‐point scale, where higher scores indicate that a document is easier to understand. Similarly, the Flesch–Kincaid Grade Level Test rates text on a “grade level” used in schools in the USA.

#### 2.3.2 Convergent Validity

To assess convergent validity, the sum scores of the EDE‐Q7 subscales were assessed using nonparametric (Spearman's *ρ*) tests with disordered eating behaviors (derived from the behavioral items of the EDE‐Q). It was expected that significant correlations would be observed, with small effect sizes (e.g., Jenkins and Davey 2020); a sample size of at least 153 was therefore required (calculated using G*Power; Faul et al. 2007).

#### 2.3.3 Confirmatory Factor Analysis

All items subjected to CFA were drawn from the 22 attitudinal items of the EDE‐Q to test the EDE‐Q7 and other models summarized in Table 1. The R package *lavaan* (version 0.6‐9; Rosseel 2012) was used for CFA, *psych* (Revelle 2021) to assess internal consistency, and *MVN* (4.0.5; Korkmaz et al. 2014) to assess multivariate normality. Given guidance of a minimum 10:1 subject‐to‐item ratio or an *N* of at least 200 (e.g., Li 2016), we aimed for a sample size of at least 220, allowing for an additional 25% (i.e., around 275 in total) in light of potential incomplete responses (e.g., Kelly et al. 2017). Given the strong factor loadings anticipated (e.g., Penelo et al. 2013; Sahlan et al. 2023), sample sizes smaller than this may be sufficient (Wolf et al. 2013).

The presence of non‐normality in the EDE‐Q was suggested by Mardia's Test, with significant skew and kurtosis, so robust estimation (Weighted Least Squares Means‐ and Variance‐adjusted; WLSMV) was used in CFA. Indicator variables (items of the EDE‐Q) were treated as categorical (using the “Ordered” function in *lavaan*; Rosseel 2012) and, to identify each model, the residual variances of each latent variable were fixed to 1 and allowed to correlate (e.g., Bollen 1989).

To assess structural validity, a range of fit indices (comparative, absolute, and parsimony correction; e.g., see Brown 2015) were used to assess global model fit, and interpreted based on the pattern of indices rather than adhering to a single cutoff. The comparative fit index (CFI), Tucker–Lewis index (TLI), standardized root mean squared residuals (SRMR), and root mean square error of approximation (RMSEA, including 90% confidence intervals) statistics are reported (see Brown 2015, and Hu and Bentler 1999). Conventional cutoffs to indicate acceptable and good fit are reported, specifically considering CFI and TLI values exceeding 0.90 as good and 0.95 as excellent, and SRMR values < 0.06 as desirable and < 0.08 as acceptable (see Hu and Bentler 1999). Less weight was attached to the results from the RMSEA given recent work suggesting that this index is likely to be affected (and indicate poorer fit) by large factor loadings (λs around 0.80; see Shi et al. 2019); therefore, whilst values < 0.10, and preferably < 0.06, are commonly recommended (e.g., Hu and Bentler 1999), values exceeding these cutoffs were not necessarily taken to indicate poor model fit, and were considered alongside other indices given, for instance, the different ways in which fit is evaluated by different indices (e.g., see Lai and Green 2016). We did not consider making modifications to individual models (such as modification indices, e.g., see Hutchinson 1998) as the aim was to investigate the fit of previously established models in this sample. To assess internal consistency reliability, the omega coefficient was used, using the *MBESS* package (Kelley 2017). Although there is no ‘gold standard’ for the interpretation of reliability, omega values exceeding 0.7 are generally considered adequate, reflecting a contribution of less than 30% of the variance of the latent variable toward the total error variance (Cheung et al. 2024).

## 3 Results

### 3.1 Readability

The readability of the EDE‐Q7 (at 199 words across 21 sentences) was estimated at 74.8 on the Flesch Reading Index (100‐point scale) and 5.2 (corresponding to 5th grade, or elementary school in the USA) on the Flesch–Kincaid Grade Level. These estimates suggest that the EDE‐Q7 is fairly easy to read with a ‘surface‐level readability’ that is appropriate for most young people over the age of 11.

### 3.2 Structural and Convergent Validity

Findings relating to the fit of each of the models tested are detailed below and in Table 3.

**TABLE 3:** Fit statistics and internal consistency reliability (Omega) of the EDE‐Q models evaluated in the study.

Authors	*χ* ^2^	df	CFI	TLI	SRMR	RMSEA (90% CIs)	Omega
Suggested cutoff	—	—	≥ 0.95 (good) ≥ 0.90 (acceptable)	≥ 0.95 (good) ≥ 0.90 (acceptable)	≤ 0.06 (good) ≤ 0.08 (acceptable)	≤ 0.06 (good) ≤ 0.10 (acceptable)	0.70–0.90 desirable
“Original”, Fairburn and Beglin (2008)	Did not converge						
“EDE‐Q7”, Grilo et al. (2013)	77.17	11	0.95	0.91	0.037	0.194 (0.149–0.241)	Dietary Restraint = 0.89 Overvaluation of Weight and Shape = 0.95 Body Dissatisfaction = 0.90
“Dufour”, Dufour et al. (2025)	90.44	5	0.79	0.59	0.061	0.543 (0.424–0.672)	0.96
“Friborg”, Friborg et al. (2013)	770.76	203	0.82	0.79	0.053	0.165 (0.151–0.179)	S1 = 0.97 S2 = 0.91 S3 = 0.90 S4 = 0.77
“Lewis‐Smith”, Lewis‐Smith et al. (2021)	250.49	53	0.90	0.88	0.051	0.149 (0.129–0.171)	Preoccupation = 0.90 Weight and Shape Concerns = 0.91
“Penelo”, Penelo et al. (2013)	994.16	208	0.78	0.75	0.063	0.181 (0.168–0.194)	Restraint = 0.91 Eating, Weight, Shape Concerns = 0.97
“Peterson”, Peterson et al. (2007)	813.78	206	0.83	0.81	0.056	0.159 (0.146–0.172)	Weight and Shape Concerns = 0.97 Eating Concern = 0.93 Dietary Restraint = 0.89
“White”, White et al. (2014)	949.25	206	0.77	0.74	0.062	0.185 (0.171–0.200)	Shape and Weight Concerns = 0.96 Restriction = 0.92 Preoccupation and Eating Concern = 0.91

Abbreviations: CFI, Comparative Fit Index; CI, confidence interval; RMSEA, root mean squared error of approximation; SRMR, standardized root mean squared residual; TLI, Tucker‐Lewis Index.

#### 3.2.1 Seven‐Item EDE‐Q [“EDE‐Q7”] (Grilo et al. 2013)

Several indices suggested good fit of this 7‐item model, although the RMSEA was above suggested cutoffs. Factor loadings were stronger than, albeit with a similar pattern to, those in previous work with clinical samples (e.g., Bang et al. 2023; Machado et al. 2018), and the three latent factors covaried to a moderate degree, often less strongly than other models studied here (i.e., ψ_(Body Dissatisfaction, Dietary Restraint)_ = 0.704; ψ_(Overvaluation of Weight and Shape, Dietary Restraint)_ = 0.739; ψ_(Overvaluation of Weight and Shape, Body Dissatisfaction)_ = 0.892). The EDE‐Q7 showed significant correlations (*p*s < 0.05) with frequency of binge eating, self‐induced vomiting, laxative use, and compulsive exercise (see Table S1). Effect sizes ranging from small (Dietary Restraint—Laxative Use) to large (Dietary Restraint—Compulsive Exercise) were seen, with the exception of the correlation between Dietary Restraint and number of days engaged in binge eating in the last month (*p* = 0.078).

### 3.3 Full‐Item Models

#### 3.3.1 “Original”

The “Original”, Fairburn and Beglin (2008) model did not converge, a problem usually associated with misspecification or “serious logical problems” (Newsom et al. 2023, 494) with the model. In common with several previous studies, the covariance between the *Shape Concern* and *Weight Concern* factors was estimated at 1 (i.e., “perfect” covariance; e.g., Allen et al. 2011) and some factor loadings were very high (e.g., Item 12 [“Have you had a strong desire to lose weight?”] on the *Weight Concern* factor; *λ* = 0.962).

#### 3.3.2 “Friborg”

This model (Friborg et al. 2013) demonstrated poor fit on all indices except for the SRMR. There was a variable pattern of factor loadings (e.g., ranging from λs of 0.434–0.907 for factor S4) and relatively high covariances with Factor S4, in particular (e.g., *ψ* = 0.912 with S2, 0.921 with S1), suggesting some redundancy.

#### 3.3.3 “Penelo”

Indices suggested poor fit of this model (Penelo et al. 2013), with variable factor loadings for both *Restraint* (e.g., Item 5 [“Have you had a definite desire to have an empty stomach with the aim of influencing your shape or weight?”], *λ* = 0.989) and *Eating, Weight, Shape Concerns* (Item 19, *λ* = 0.412; Item 12, *λ* = 0.956).

#### 3.3.4 “Peterson”

Most fit indices indicated poor fit of the model (Peterson et al. 2007). Additionally, variable item loadings were seen: Item 19, for example, showed a low factor loading (of 0.429) on the *Eating Concern* factor, which is similar in content to that of White et al.'s *Preoccupation* factor.

#### 3.3.5 “White”

Most fit indices (see Table 3) suggested poor fit of White et al. (2014) model. Factor loadings of several items (e.g., Item 10 [“Have you had a definite fear that you might gain weight?”] on the *Restriction* factor were very high (*λ* = 0.994)), and others were low (e.g., Item 19 [“How many days have you eaten in secret?”] on the *Preoccupation* factor; *λ* = 0.432).

### 3.4 Reduced‐Item (“Brief”) Models

#### 3.4.1 “Dufour”

This one‐factor model (Dufour et al. 2025) evidenced poor fit. Strong loadings (all > 0.91) of all items suggest item redundancy or poor content validity.

#### 3.4.2 “Lewis‐Smith”

Results suggested acceptable fit of this model (Lewis‐Smith et al. 2021) on several fit indices although, in common with all models tested, the RMSEA was above the recommended cutoff. The two factors of this model were correlated at 0.926, indicating a large amount of shared variance and poor discriminant validity. There was an acceptable range of factor loadings (λs ranging from 0.721 to 0.924 for *Preoccupation*, and 0.681–0.942 for *Weight and Shape Concern*).

### 3.5 Internal Consistency Reliability

Omega values are reported in Table 3, with all scales and subscales, including those of the EDE‐Q7, suggestive of acceptable internal consistency reliability.

## 4 Discussion

The principal aim of this study was to evaluate the readability and structural validity of a brief version of the EDE‐Q—the EDE‐Q7 (Grilo et al. 2013)—in a clinical sample of adolescents having a diagnosed eating disorder. In line with recent studies of young people (Sahlan et al. 2023; Trujillo‐ChiVacuán et al. 2025), acceptable model fit across several indices was found, suggesting that the EDE‐Q7 may offer valid assessment of disordered eating attitudes in adolescents, including those presenting to clinical settings. Other studies examining the factor structure of the EDE‐Q in adolescent samples have supported the EDE‐Q7 over alternatives, including some models evaluated here (e.g., Trujillo‐ChiVacuán et al. 2025), although there remains variability in findings regarding the structural validity of the EDE‐Q in adolescents (e.g., Forsén Mantilla et al. 2017).

Taking existing work alongside the current study, the EDE‐Q7 offers a brief assessment of eating psychopathology that has shown structural validity in samples from 13 years of age. By contrast, there was little support in this study for models comprising all 22 attitudinal items of the EDE‐Q, in line with similar work in transdiagnostic ED samples (e.g., Allen et al. 2011; Calugi et al. 2017). The two‐factor model of Lewis‐Smith et al. (2021), comprising 12 items and derived from a sample of secondary school students in India, showed a comparable pattern of model fit. Whilst the additional items contained in the “Lewis‐Smith” model might allow for a broader assessment of ED symptoms, the two subscales (“Preoccupation” and “Weight and Shape Concerns”) were found to be strongly correlated and different versions of the measure may be required across genders. A study of Iranian adolescents and university students (Sahlan et al. 2023) provided psychometric support for use of the EDE‐Q7, but failed to find evidence supporting the structural validity of the “Lewis‐Smith” model, suggesting that it might not generalize to other cultural settings.

Supporting psychometric assessment of the EDE‐Q7, the estimated reading grade level suggests that the text would be read effectively by most individuals aged 11 and older, and potentially by younger adolescents as well. Although adaptations of the EDE‐Q for use in younger samples have been suggested (e.g., Carter et al. 2001), this brief version may be appropriate in both epidemiological studies and clinical work where a valid, short assessment is required, or where mixed adolescent/adult samples are included. Further advantages of having a measure, such as the EDE‐Q7, that is usable across different samples include the possibility of determining measurement invariance—an early step in the accurate comparison of ED symptoms across different groups, such as age, gender, and diagnosis (e.g., Peláez‐Fernández et al. 2025). In light of growing evidence that the EDE‐Q7 consistently shows structural validity, this study supports a call for more widespread adoption of this measure in the ED field (Reilly et al. 2025).

Our findings, whilst based on a sample with a large proportion of female participants and those diagnosed with anorexia nervosa or atypical anorexia nervosa, also add to existing data on the structural validity of the EDE‐Q in adolescent and adult samples (see Jenkins and Rienecke 2022, for a review). Consistent failure to support the factor structure of the ‘Original’ version suggests that revision of the scoring may be needed, and the current study provides further support for a brief version which can be extracted from the longer EDE‐Q or used as a 7‐item “standalone” measure (e.g., Jenkins and Davey 2020). Further analyses are needed to appraise the utility of the EDE‐Q7, and perhaps other shortened versions, for assessing treatment outcomes and the ability to model change in eating disorder symptoms over time, including when used as a standalone measure. The findings presented here also support the convergent validity of the EDE‐Q7. Reflecting work in adult samples (e.g., Jenkins and Davey 2020), the EDE‐Q7 showed consistent correlations with disordered eating behaviors, supporting other assessments of convergent validity in adolescents (e.g., Penelo et al. 2013; Trujillo‐ChiVacuán et al. 2025).

The findings should be understood in the context of all models exceeding cutoffs (values > 0.10) on a measure of absolute fit—the RMSEA—a pattern which has emerged in other studies of the EDE‐Q (e.g., Allen et al. 2011), as well as similar measures such as the ED‐15 (e.g., Zhou et al. 2024). This is likely to be explained by the generally strong factor loadings seen within the EDE‐Q and the focus of the RMSEA on mis‐specified loading patterns (Goretzko et al. 2023); this was perhaps most evident in the five‐item model of Dufour et al. (2025) as all items loaded on the factor above 0.90. Model fit indices showed a mixed pattern regarding the EDE‐Q7, with CFI and SRMR indicating good fit, and RMSEA suggesting poor fit. As detailed by Lai and Green (2016), such discrepancies are not uncommon in CFA models, even for models that are not obviously mis‐specified, and are particularly likely when factor loadings are strong. As such, conclusions regarding model fit must consider the context of the overall pattern of evidence rather than relying on any single cutoff (see also Goretzko et al. 2023). Future studies using CFA to evaluate the structural validity of the EDE‐Q should consider this finding, and evaluate model fit with that in mind. Estimates of internal consistency all fell within acceptable limits of reliability, although should be interpreted with caution for models with poor fit, given that omega “can be biased if its underlying model is mis‐specified” (Green and Yang 2015, 19).

By including a clinical adolescent sample, the current study advances findings in community (e.g., Penelo et al. 2013) and mixed (e.g., Forsén Mantilla et al. 2017) samples by further supporting the EDE‐Q7 and evaluating other models, including those with support in adult samples (e.g., Friborg et al. 2013). As a briefer measure, the EDE‐Q7 may be desirable when resources (e.g., time) are scarce and is bolstered by omission of items that may be less useful in certain populations (e.g., see Darcy et al. 2013) and a lower estimated reading age level. However, this brevity also limits scope and risks omitting symptoms, experiences, or latent constructs which are important to patients and outcome measurement. Therefore, studies designed to fully explore the risks and benefits associated with briefer forms of the EDE‐Q are needed. The current findings suggest that the EDE‐Q7 might be useful in samples of both adolescents and adults given evidence of structural validity and greater readability compared to the 22‐item version. As such, whilst more thorough assessments of reading and comprehension are needed to assess measure complexity and depth of understanding (e.g., Richards et al. 2013), it is important to further establish the longitudinal stability of the EDE‐Q7 and sensitivity to treatment effects in adolescents, given that there is emerging evidence that the EDE‐Q can discriminate between patients and comparisons (e.g., Bang et al. 2023; Machado et al. 2018) and capture changes as a result of treatment (e.g., Machado et al. 2020).

### 4.1 Strengths and Limitations

The current sample made use of robust assessments of fit and several different indices to evaluate fit in the context of model complexity (e.g., see Lai and Green 2016). Recruitment from different countries provides some support for use of the EDE‐Q7 across cultural contexts and further assessment is indicated. The sample size was sufficiently large for CFA, although elements of complexity (e.g., non‐normality, relatively few indicators per factor) may affect the “ideal” sample size (see Wolf et al. 2013). Whilst the clinical sample included a range of ED diagnoses, it was characterized by a high proportion of female adolescents and those diagnosed with anorexia nervosa, which may limit generalisability and render the findings more representative of individuals presenting with low weight. Furthermore, diversity of gender, race, and ethnicity in the sample was limited, although a study of a large community sample suggests that the EDE‐Q7 can be used to assess eating disorder symptomatology in gender minority populations (Nagata et al. 2023). The lack of assessment of other aspects of eating psychopathology (e.g., body image; see Sahlan et al. 2023) was a limitation, and test–retest reliability was not assessed in the current study (but see Penelo et al. 2013). Use of the behavioral items of the EDE‐Q to assess convergent validity may have limited the strength of the validity evidence, and future work should consider existing limitations related to establishing construct validity in ED research, including providing greater evidence of discriminant validity (see Forbush et al. 2017).

Future investigations of the EDE‐Q and its items might also consider other influences on its usefulness, including adolescents' conceptual understanding and interpretation (e.g., see Parker et al. 2023), and include broader assessments of reliability and validity, including qualitative methods (e.g., Huellemann et al. 2026). Comparison of EDE‐Q7 subscale and Total scores across different samples (e.g., by diagnosis) would be useful, in addition to consideration of the longitudinal stability of the EDE‐Q7 in adolescents, which could address both psychometric and clinical questions, including whether the latent variables of the EDE‐Q7 might have a hierarchical measurement structure.

### 4.2 Conclusions

The principal hypothesis of this study—that the EDE‐Q7 would show structural validity in a sample of adolescents diagnosed with eating disorders—was confirmed. There was limited support for the structural validity of models using all available attitudinal items of the EDE‐Q (including the ‘Original’ version). The current study, with a sample of adolescents recruited from different clinical settings in two different countries, adds to growing evidence that the EDE‐Q7 consistently shows structural validity and represents a valid, brief assessment of eating psychopathology across the age range.

## Author Contributions

**Renee D. Rienecke:** writing – review and editing, conceptualization, methodology. **Elizabeth K. Hughes:** writing – review and editing. **Sarah Eckhardt:** writing – review and editing. **Erin C. Accurso:** writing – review and editing. **Daniel Le Grange:** conceptualization, writing – review and editing. **Sasha Gorrell:** data curation, formal analysis, methodology, writing – review and editing, writing – original draft. **Paul E. Jenkins:** conceptualization, data curation, formal analysis, writing – original draft, writing – review and editing, methodology. **Susan M. Sawyer:** writing – review and editing.

## Lived Experience Involvement Statement

No specific efforts were undertaken to involve persons with lived experience in the study design or execution, or in the preparation of this manuscript.

## Funding

Funding support for data collection in Australia was provided by a grant from the Baker Foundation (Australia). The Murdoch Children's Research Institute is supported by the Victorian Government's Operational Infrastructure Support Program. We also acknowledge the generous financial support from the Goven Family Foundation, who made it possible for the Children's Minnesota team to conduct this study.

## Ethics Statement

This study was conducted in accordance with the Declaration of Helsinki. The Institutional Review Boards at each participating institution gave approval for the ongoing use of data within data use agreements. Informed consent was obtained from the participants' parent or guardian. Persons with lived experience were not involved in the study design or execution, or in the preparation of this manuscript.

## Conflicts of Interest

Dr. Le Grange receives royalties from Routledge and Guilford press, he is co‐director of the Training Institute for Child and Adolescent Eating Disorders LLC, and is a member of the Clinical Advisory Board of Univa Health Ltd. Dr. Rienecke receives consulting fees from the Training Institute for Child and Adolescent Eating Disorders LLC, and receives royalties from Routledge and Hachette Book Group Inc.

## Supporting information


**Table S1:** Correlations between EDE‐Q7 subscales (Grilo et al. 2013) and disordered eating behaviors (taken from the EDE‐Q 6.0; Fairburn and Beglin 2008).

## Data Availability

Deidentified data are available upon reasonable request by e‐mailing Dr. Sasha Gorrell (sasha.gorrell@ucsf.edu).
