# Supplementary material for: Structural Validity of a Short Form of the Eating Disorder Examination Questionnaire in Adolescents Diagnosed With an Eating Disorder
Source: Int J Eat Disord. 2026 Jun 15;59(10):2196–206. doi: 10.1002/eat.70146 (PMC13766238; doi:10.1002/eat.70146)
Supplement: Supplementary file 1 [file EAT-59-2196-s001.docx]

**Supplementary Table 1**

Correlations between EDE-Q7 subscales (Grilo et al., 2013) and disordered eating behaviours (taken from the EDE-Q 6.0; Fairburn & Beglin, 2008.

| Variable | Overvaluation of Weight and Shape | Body Dissatisfaction | OBE Frequency^†^ | OBE days^‡^ | Self-induced vomiting | Laxative use | Compulsive exercise |
| --- | --- | --- | --- | --- | --- | --- | --- |
| Dietary Restraint | .62** | .56** | .15* | .11 | .20** | .14** | .51** |
| Overvaluation of Weight and Shape | - | .78** | .20** | .19** | .31** | .23** | .29** |
| Body Dissatisfaction | - | - | .26** | .26** | .40** | .28** | .28** |

Note. *N*s = 248 – 258; OBE = Objective Binge Eating; ^†^Number of times in last 28 days; ^‡^Number of days in last 28; *p < .05. **p < .01.
